# The long shadow of childhood trauma for depression in midlife: examining daily psychological stress processes as a persistent risk pathway

**DOI:** 10.1017/S0033291721000921

**Published:** 2022-12

**Authors:** Stefanie E. Mayer, Agus Surachman, Aric A. Prather, Eli Puterman, Kevin L. Delucchi, Michael R. Irwin, Andrea Danese, David M. Almeida, Elissa S. Epel

**Affiliations:** 1 Department of Psychiatry and Behavioral Sciences, University of California, San Francisco, CA, USA; 2 Department of Human Development and Family Studies, The Pennsylvania State University, University Park, PA, USA; 3 Center for Healthy Aging, The Pennsylvania State University, University Park, PA, USA; 4 School of Kinesiology, University of British Columbia, Vancouver, BC, Canada; 5 Cousins Center for Psychoneuroimmunology, UCLA Semel Institute for Neuroscience and Human Behavior, Los Angeles, CA, USA; 6 Social, Genetic and Developmental Psychiatry Centre and Department of Child and Adolescent Psychiatry, Institute of Psychiatry, Psychology and Neuroscience, King's College London, London, UK; 7 National and Specialist CAMHS Clinic for Trauma, Anxiety, and Depression, South London and Maudsley NHS Foundation Trust, London, UK

**Keywords:** Childhood trauma, early life adversity, depression, daily stress, daily diaries, stress appraisals, persistent risk pathway

## Abstract

**Background:**

Childhood trauma (CT) increases the risk of adult depression. Buffering effects require an understanding of the underlying persistent risk pathways. This study examined whether daily psychological stress processes – how an individual interprets and affectively responds to minor everyday events – mediate the effect of CT on adult depressive symptoms.

**Methods:**

Middle-aged women (*N* = 183) reported CT at baseline and completed daily diaries of threat appraisals and negative evening affect for 7 days at baseline, 9, and 18 months. Depressive symptoms were measured across the 1.5-year period. Mediation was examined using multilevel structural equation modeling.

**Results:**

Reported CT predicted greater depressive symptoms over the 1.5-year time period (estimate = 0.27, s.e. = 0.07, 95% CI 0.15–0.38, *p* < 0.001). Daily threat appraisals and negative affect mediated the effect of reported CT on depressive symptoms (estimate = 0.34, s.e. = 0.08, 95% CI 0.22–0.46, *p* < 0.001). Daily threat appraisals explained more than half of this effect (estimate = 0.19, s.e. = 0.07, 95% CI 0.08–0.30, *p* = 0.004). Post hoc analyses in individuals who reported at least moderate severity of CT showed that lower threat appraisals buffered depressive symptoms. A similar pattern was found in individuals who reported no/low severity of CT.

**Conclusions:**

A reported history of CT acts as a latent vulnerability, exaggerating threat appraisals of everyday events, which trigger greater negative evening affect – processes that have important mental health consequences and may provide malleable intervention targets.

## Introduction

Traumatic childhood experiences (physical, sexual, emotional abuse, and neglect) can leave ‘scars’ on adult life – increasing risk for mental health disorders, including depression (Green et al., 2010; Kendler et al., 2000; Kessler, Davis, & Kendler, 1997). Buffering these effects requires an understanding of the underlying risk pathways. Daily cognitive-affective stress processes have been proposed as one pathway linking childhood trauma (CT) to adult health (Epel et al., 2018; Miller, Chen, & Parker, 2011). However, this has not been directly tested. Deepening our understanding of the daily psychological mechanisms can inform non-pharmacological interventions.

Individuals’ psychological responses to daily stressors can impact health (Charles, Piazza, Mogle, Sliwinski, & Almeida, 2013; Epel et al., 2018; Lazarus, 1999). The perceived negative impact of the stressor, termed cognitive threat appraisal (Lazarus, 1999), determines whether the event is interpreted as a threat or challenge (Blascovich & Mendes, 2010). Threat appraisals also mold affective stress responses (Blascovich & Mendes, 2010; Lazarus, 1999). CT shapes individuals' habitual ways of appraising and affectively responding to everyday stressors, as shown in daily diary studies (Glaser, van Os, Portegijs, & Myin-Germeys, 2006; Infurna, Rivers, Reich, & Zautra, 2015; Kong, Martire, Liu, & Almeida, 2019; Lardinois, Lataster, Mengelers, Van Os, & Myin-Germeys, 2011; Mallers, Charles, Neupert, & Almeida, 2010; Weltz, Armeli, Ford, & Tennen, 2016). For example, more frequent experiences of reported childhood abuse are associated with greater threat appraisals and negative affect in response to daily adult stressors (Kong et al., 2019). Daily stress processes, in return, shape the extent to which daily events have the potential to impact mental and physical health. For example, threat appraisals in response to laboratory stress are associated with shorter TL (O'Donovan et al., 2012) and greater inflammation (Slavich & Irwin, 2014). Similarly, negative affect in the evening after a minor daily event predicts risk for depression (Charles et al., 2013; Cohen, Gunthert, Butler, O'Neill, & Tolpin, 2005), chronic physical health problems (Piazza, Charles, Sliwinski, Mogle, & Almeida, 2013), and systemic inflammation (Sin, Graham-Engeland, Ong, & Almeida, 2015) years later.

Overall, daily cognitive-affective stress processes appear to be a highly plausible psychological pathway linking CT to increased depression risk. This study directly tested the mediational pathway in a sample of healthy women who completed daily stress diaries along with self-report measures of CT and depressive symptoms. We hypothesized that reported CT will predict higher depressive symptoms, which will be mediated by more maladaptive daily stress processes (greater threat appraisals and greater negative affect).

## Methods

### Participants

Participants were 183 mothers from a longitudinal study that examined the impact of chronic caregiver stress on biological markers of stress, cellular aging, and wellbeing. Participants were recruited via social media and local community advertisements (e.g. schools, parenting publications, child development centers in the San Francisco Bay Area). Eligible participants were 20–50 years old, premenopausal, non-smoking, with no major physical diseases (including no history of coronary heart disease, endocrine disorders, epilepsy, brain injury, autoimmune conditions, severe asthma or lung disease), and had at least one child between the ages of 2 and 16 years. Maternal caregivers (*n* = 92) had to care for a child diagnosed with autism spectrum disorder and report a score of ⩾13 on the Perceived Stress Scale (PSS; Cohen, 1988; Cohen, Kamarck, & Mermelstein, 1983). Maternal controls (*n* = 91) had to care for a neurotypical child and report a score of ⩽19 on the PSS. The PSS criteria allowed for analyses across the continuum of perceived chronic stress, independent of caregiver group status. Participants were excluded if they had a current psychiatric condition as determined by questions from the Structured Clinical Interview for Diagnostic and Statistical Manual for Mental Disorders for Axis I Disorders (SCID), including bipolar disorder, posttraumatic stress disorder and eating disorders, and, for maternal controls, current major depression. Current major depression and antidepressant use were permitted among caregivers because depression is a common response to chronic stress. We chose this sample to examine links between CT, daily stress processes, and depressive symptoms because the study recruited participants across the stress and depression spectrum, with one-third of the sample reporting at least moderately severe CT. Another strength of the study was that rich daily diary data were collected over a period of 1.5 years, allowing us to capture habitual responses to everyday stressors across different life circumstances.

### Overview of procedures

During a laboratory baseline assessment, participants provided informed consent and self-report measures. They also completed 7 days of daily diaries. Procedures were repeated at 9, 18, and 24 months. Forty-three percent of participants completed a mindfulness intervention between 18 and 24 months, limiting current analyses to the initial 1.5 years. The study was approved by the local Institutional Review Board.

### Self-report measures

#### Sociodemographic information

Age, race, marital status, education, annual household income, and caregiver group status (0 = maternal controls; 1 = maternal caregivers) were assessed at baseline.

#### Childhood trauma

CT was assessed via retrospective self-report at baseline with the Childhood Trauma Questionnaire (CTQ; *α* = 0.84; Bernstein & Fink, 1998). CTQ subscales were calculated for emotional abuse (*α* = 0.85), physical abuse (*α* = 0.73), sexual abuse (*α* = 0.93), emotional neglect (*α* = 0.90), and physical neglect (*α* = 0.61). Main analyses treated CT as a latent variable based on CTQ subscales – a measurement model that fulfilled overall goodness of fit criteria in a confirmatory factor analysis (RMSEA = 0.06, CFI = 0.99, TLI = 0.96, SRMR = 0.01). The standardized factor loadings ranged from 0.32 to 0.83, exceeding the widely-used cutoff of 0.30 (Floyd & Widaman, 1995). Post hoc analyses defined CT categorically, as at least moderate severity in at least one CTQ subscale (see CTQ manual; Bernstein & Fink, 1998).

#### Depressive symptoms

Depressive symptoms were assessed using the self-report version of the 30-item Inventory of Depressive Symptomatology (IDS-SR; Rush *et al*., 1986; Rush, Carmody, & Reimitz, 2000; Rush, Gullion, Basco, Jarrett, & Trivedi, 1996) at baseline (Cronbach's *α* = 0.82), 9 months (*α* = 0.87), and 18 months (*α* = 0.88). Participants are asked to rate the severity and frequency of specific symptoms present over the last 7 days. Items are rated on a four-point scale (0–3) with variable response options. The total score (sum of all items) is based on 28 items due to branching options (either decreased or increased appetite, but not both; either increased or decreased weight, but not both). The total score ranges from 0 to 84 with higher scores indicating greater depressive symptom severity. Total IDS-SR scores can be interpreted as none (0–13), mild (14–25), moderate (26–38), severe (39–48), and very severe (49–84) depressive symptoms (Rush et al., 2003; Trivedi et al., 2004). The IDS-SR has a good construct validity (Rush et al., 1996). Depressive symptoms were highly stable over the 1.5-year time span (see online Supplementary Fig. S1; Cronbach's *α* over time = 0.89), so values were averaged to create a single outcome measure.

#### Perceived stress

Perceived stress over the past month was assessed at baseline with the 10-item PSS (*α* = 0.87; Cohen, 1988; Cohen *et al*., 1983). It served as a covariate in sensitivity analyses to control for current overall perceived stress at the time of CT recall.

### Daily diaries

Participants completed daily evening diaries for 7 days at baseline, 9, and 18 months.

#### Daily stressors

Participants described ‘the event in your life that caused you the most stress today’. Descriptions were objectively coded for stressor severity by two independent research assistants (for details, see Almeida, Wethington, & Kessler, 2002; Catalino, Arenander, Epel, & Puterman, 2017; Crosswell, Coccia, & Epel, 2020). Days with no (objectively coded) stressors (3%) were rare and excluded from analyses. Events of low severity occurred on 51% of days and events of moderate severity on 37%. Events of high (8%) and extreme (<1%) severity were less frequent, so stressor days of at least moderate severity (objectively coded) were combined into one category. A person's average exposure to stress days that were objectively coded as being of at least moderate severity served as a covariate. This ensured that subjective ratings of threat appraisals and negative affect in response to daily events were not a result of the fact that participants differed in the degree to which they actually lived more or less stressful lives (higher/lower average number of days over the past 1.5 years that were objectively considered as ‘at least moderately stressful’ by two independent raters).

#### Threat appraisals

Threat appraisals were measured using items from the Daily Inventory of Stressful Events (DISE; Almeida et al., 2002). First, participants indicated whether the stressful situation had a negative effect on any of the eight appraisal domains: ‘Today, did this stressful situation have a negative effect on: (1) your daily routine, (2) your financial situation, (3) the way you feel about yourself, (4) the way others feel about you, (5) your physical health or safety, (6) the health or well-being of someone you care about, (7) your plans for the future, (8) your relationship with someone close to you.’ Participants checked all domains that applied (response options: selected or not). Then, for each domain that was selected, a follow-up question assessed the subjective severity of the negative impact on that domain: ‘How much did this stressful situation negatively impact [the selected domain]’. Responses were assessed on a four-point scale (1 = ‘A little bit’; 4 = ‘A lot’). A single threat appraisal variable was calculated by summing all subjective severity ratings across all selected domains and dividing it by the number of selected domains. The measure thus reflects the average threat appraisal severity across selected domains. Subjective severity and appraisal domain items have been shown to correlate with daily physical symptoms and daily negative mood (Almeida et al., 2002).

#### Negative affect

Participants reported their current (evening) affect using the modified Differential Emotions Scale (mDES; Fredrickson, Tugade, Waugh, & Larkin, 2003). A single variable was computed by averaging the 12 negative valence items.

### Statistical analysis

To examine mediation, we used a multilevel structural equation modeling (MSEM) framework to take into account nested (daily diary) data while also testing path models (for details, see Heck & Thomas, 2015; Mehta & Neale, 2005; Surachman, Wardecker, Chow, & Almeida, 2019). Analyses were conducted using M*plus* version 8.1 (Muthén & Muthén, 2012). We used the robust maximum likelihood estimation with robust standard errors to deal with missing data. To test the hypothesized model (see online Supplementary Fig. S2), we first tested a structural equation model of the association between reported CT and depressive symptoms. The structural equation model was then contrasted to a two-level MSEM finding where we examined whether daily stress processes mediated the association between reported CT and depressive symptoms. The level-1 (within-level) model focused on the prediction of daily negative evening affect by daily threat appraisal. The level-2 (between-level) model focused on the associations between the latent predictor of CT, the latent means of threat appraisals and negative evening affect (latent means provide a more precise estimation of individuals' true means than observed aggregates; Ludtke et al., 2008; Ludtke, Marsh, Robitzsch, & Trautwein, 2011; Marsh et al., 2009), and the observed outcome of depressive symptoms. Finally, indirect effects examined the mediating role of daily stress processes on the association between reported CT and depressive symptoms (Muthén & Muthén, 2012). Sensitivity analyses added the PSS to the MSEM model to at least partly account for the possibility that current overall perceived stress in the past month may introduce biases in retrospective self-reports of CT (Danese & Widom, 2020). Moreover, we compared the hypothesized model to an alternative model, in which depressive symptoms mediated the association between CT and daily stress processes (see online Supplementary Fig. S3). Lastly, we conducted exploratory models within caregivers and controls separately. The estimates reported are based on the standardized results (for unstandardized estimates, please refer to the Supplementary Material) .

#### Missing data

Missing data are minimal in the data set. Most of the missing data are related to information regarding CTQ, ranging from 7 (CTQ emotional neglect) to 12 (CTQ sexual abuse). Little's MCAR test for CTQ variables indicated that data were missing at random [χ^2^ (df = 35) = 21.88, *p* = 0.96]. Furthermore, our analyses utilized maximum likelihood estimation with robust standard errors that is known to be robust for handling missing data and non-normal distribution.

## Results

### Sample characteristics

Socio-demographic information is presented in Table 1. On average, depressive symptoms were mild, but 21% (38/182) had at least moderate depressive symptoms at least once over the 1.5-year period. About one-third of participants reported at least moderately severe scores in at least one CTQ subscale; these participants reported higher depressive symptoms over the 1.5-year period relative to those with no/low reported CT (IDS *M* = 17, s.e. = 8 *v*. *M* = 14, s.e. = 7, Cohen's *d* = 0.4), with higher mild [78% (43/55) *v.* 55% (58/105)] and moderate [31% (17/55) *v.* 17% (18/105)] depressive symptoms. Maternal caregivers reported greater daily threat appraisals, daily negative affect, and depressive symptoms across the 1.5-year period compared to maternal controls. Thus, caregiver status was a control variable in models.

**Table 1.** Descriptive statistics for sociodemographic information, childhood trauma type, daily stress responses, and depressive symptoms

		Entire sample (*n* = 183)	Caregivers (*n* = 92)	Controls (*n* = 91)
Category	Variable	Mean (s.d.) or No. (%)	Mean (s.d.) or No. (%)	Mean (s.d.) or No. (%)
Sociodemographics	Age, mean (s.d.), years	42.43 (5.09)	42.77 (5.63)	42.07 (4.49)
	Race, No. (%) Non-Hispanic White	139 (76)	70 (76)	69 (76)
	Non-Hispanic Black	6 (3)	2 (2)	4 (4)
	Non-Hispanic Asian/Pacific Islander	18 (10)	10 (11)	8 (9)
	Non-Hispanic Native American	1 (0.5)	0 (0)	1 (1)
	Non-Hispanic Other/Multiracial	5 (3)	3 (3)	2 (2)
	Hispanic or Latina	14 (8)	7 (8)	7 (8)
	Marital status, No. (%) married	158 (87)	81 (89)	77 (85)
	Education, No. (%) bachelor's degree or higher	155 (87)	74 (82)	81 (91)
	Annual household income, No. (%) ⩾$ 100 000	139 (76)*	62 (68)	77 (85)
Childhood trauma type	CTQ – Emotional Abuse Subscale, mean (s.d.)	8.36 (3.80)*	9.14 (4.17)	7.63 (3.28)
	CTQ – Physical Abuse Subscale, mean (s.d.)	6.10 (2.16)	6.29 (2.22)	5.92 (2.10)
	CTQ – Sexual Abuse Subscale, mean (s.d.)	5.81 (2.79)	6.09 (3.05)	5.52 (2.49)
	CTQ – Emotional Neglect Subscale, mean (s.d.)	10.47 (4.38)	10.98 (4.65)	9.95 (4.05)
	CTQ – Physical Neglect Subscale, mean (s.d.)	6.27 (1.87)*	6.57 (2.22)	5.98 (1.38)
Daily stress responses	Threat appraisal (mean over 1.5 years)	1.61 (0.57)*	1.81 (0.57)	1.40 (0.50)
	Negative affect (mean over 1.5 years)	0.51 (0.35)*	0.64 (0.39)	0.39 (0.24)
Depressive symptoms	IDS (mean over 1.5 years)	14.94 (7.56)*	18.70 (7.67)	11.19 (5.25)

CTQ, Childhood Trauma Questionnaire; IDS, Inventory of Depressive Symptomatology.

*Indicates significant group differences (*p* < 0.05).

### Childhood trauma and depressive symptoms

**Fig. 1.**
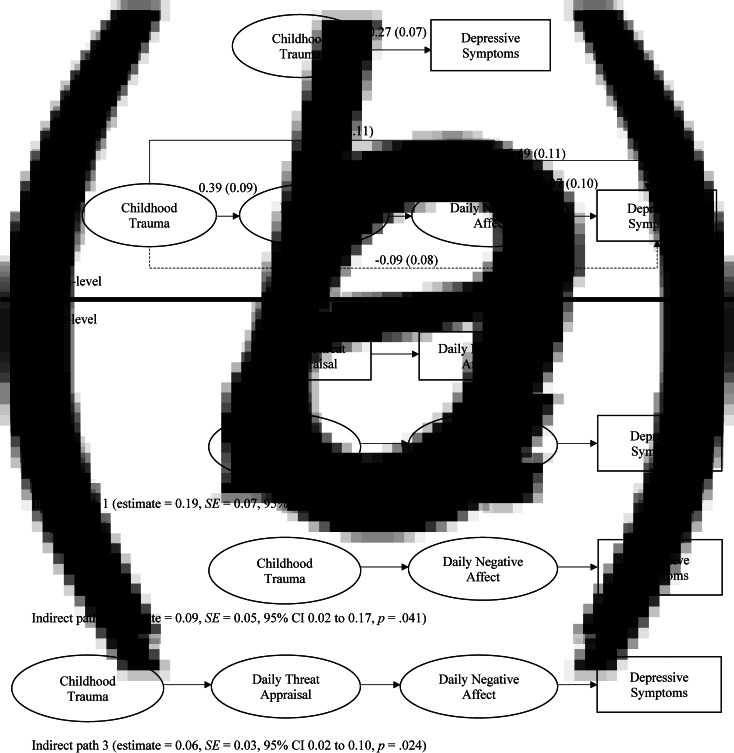
Findings from the prediction of depressive symptoms by reported childhood trauma. Greater reported childhood trauma predicted higher depressive symptom, adjusting for age, marital status, education, annual household income, and caregiver group status (*a*; structural equation model). However, after adding daily stress processes into the model (*b*; multilevel structural equation modeling, MSEM), the direct association between reported childhood trauma and depressive symptoms was no longer significant, indicating mediation by daily threat appraisals and daily negative affect. The indirect analysis indicated that there was a total indirect effect from reported childhood trauma to depressive symptoms through daily stress processes (estimate = 0.34, s.e. = 0.08, 95% CI 0.22–0.46, *p* < 0.001), in which more than 50% of the indirect effect was explained through daily threat appraisals (*c*; indirect path 1). *Note*: All reported estimates are standardized (for unstandardized estimates, see online Supplementary Table S1); squares = observed variables; circles = latent variables. MSEM analyses adjusted for age, marital status, education, income, caregiver group status, and between-person differences in exposure to at least moderately severe stress days.

The structural equation model indicated that greater reported CT predicted higher depressive symptoms over the 1.5-year period (Fig. 1a; estimate = 0.27, s.e. = 0.07, 95% CI 0.15–0.38, *p* < 0.001), controlling for age, marital status, education, annual household income, and caregiver status. The MSEM findings indicated that the majority of the variances for threat appraisal (76%) and negative affect (59%) were within-day, supporting the need to consider the nesting data. Adding daily stress processes into the model improved the goodness of fit criteria (see Table 2). Detailed results from the MSEM analysis are presented in online Supplementary Table S1, including unstandardized and standardized estimates. Findings from level-1 (Fig. 1b; within-level) indicated that on a day in which participants reported greater threat appraisals, they also reported greater negative affect in the evening (estimate = 0.28, s.e. = 0.02, 95% CI 0.25–0.31, *p* < 0.001), controlling for between-person differences in exposure to at least moderately severe stress days (objective severity codes from independent raters).

**Table 2.** Model fit indices

Model	χ^2^	df	χ^2^/df	RMSEA	CFI	TLI	SRMR-W	SRMR-B	AIC	BIC	a-BIC
Childhood trauma, daily stress processes, and depressive symptoms											
s.e.m.	76.93	42	1.83	0.08	0.90	0.86	0.07^a^		9382.8	9530.0	9384.3
Hypothesized MSEM	126.64	66	1.92	0.02	0.94	0.92	0.00	0.08	24 826.8	25 200.7	25 003.7
Alternative MSEM^b^	199.54	66	3.02	0.03	0.89	0.84	0.00	0.12	24 899.8	25 255.6	25 068.1

s.e.m., structural equation model; MSEM, multilevel structural equation model; χ^2^, chi-square test of model fit; df, degrees of freedom for the χ^2^ test of model fit; RMSEA, root mean square error of approximation; CFI, comparative fit index; TLI, Tucker–Lewis index; SRMR-B, standardized root mean square residual for between level; SRMR-W, standardized root mean square residual for within level; AIC, Akaike's information criterion; BIC, Bayesian information criterion; a-BIC, sample-size adjusted Bayesian information criterion.

a The structural equation model only has one value of standardized root mean square residual.

b Alternative MSEM with depressive symptoms as mediator.

**Fig. 2.**
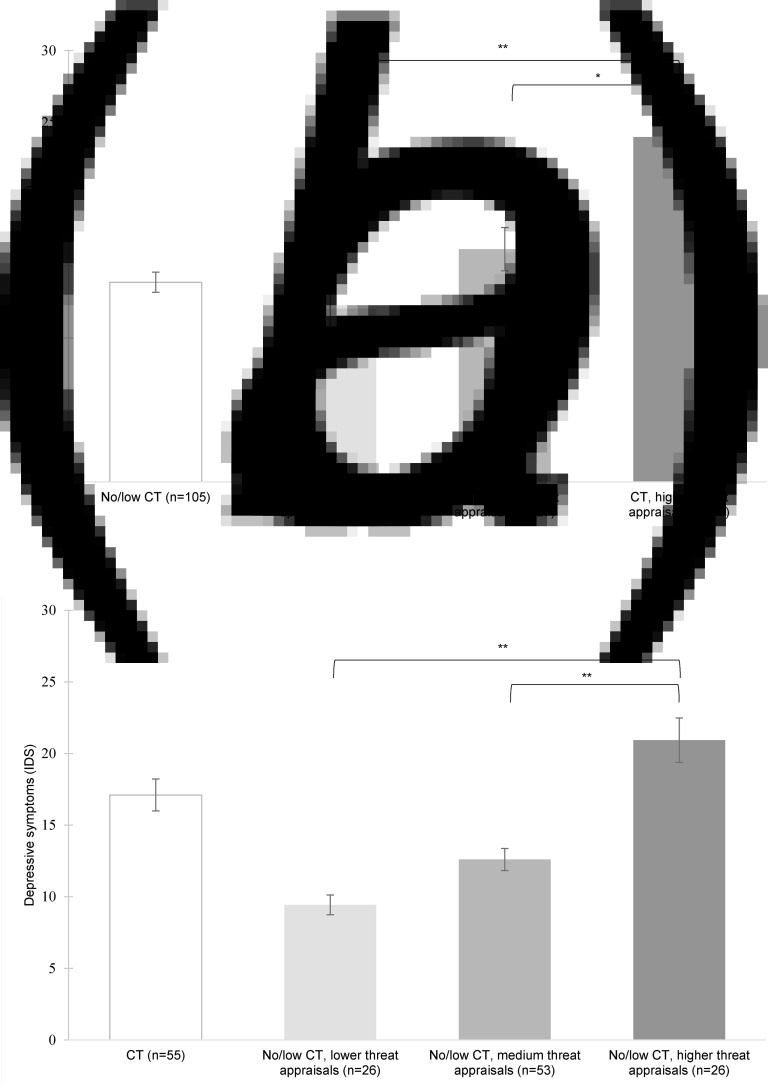
Associations between daily threat appraisals and depressive symptoms. (*a*) Depressive symptoms (Inventory of Depressive Symptomatology, IDS) among participants who reported at least moderate levels of childhood trauma (CT; defined as at least moderate severity in at least one subscale of the Childhood Trauma Questionnaire) based on their lower (⩽ 25th percentile), medium (between >25th and <75th percentile), or higher (⩾75th percentile) daily threat appraisals. Daily threat appraisals predicted depressive symptoms in individuals who reported CT (*F*_2,52_ = 10.33, *p* < 0.001). Bonferroni corrected tests showed that individuals with higher threat appraisals had higher depressive symptoms compared to individuals with medium and lower threat appraisals. Mean depressive symptoms for the no/low CT group was included as a reference. *Note*: **p < 0.001; *p < 0.01. (*b*) Depressive symptoms (Inventory of Depressive Symptomatology, IDS) among participants who reported no or low levels of childhood trauma (no/low CT) based on their lower (⩽25th percentile), medium (between >25th and <75th percentile), or higher (⩾75th percentile) daily threat appraisals. Daily threat appraisals predicted depressive symptoms in individuals who reported no/low CT (*F*_2102_ = 27.73, *p* < 0.001). Bonferroni corrected tests showed that individuals with higher threat appraisals had higher depressive symptoms compared to individuals with medium and lower threat appraisals. Mean depressive symptoms for the CT group was included as a reference. *Note*: **p < 0.001.

Results from level-2 (Fig. 1b, between-level; adjusting for age, marital status, education, income, caregiver status, and between-person differences in exposure to at least moderately severe stress days) indicated that greater reported CT predicted greater daily threat appraisals (estimate = 0.39, s.e. = 0.09, 95% CI 0.24–0.53, *p* < 0.001) and negative affect (estimate = 0.26, s.e. = 0.11, 95% CI 0.08–0.43, *p* = 0.019). These results indicated that, on average, 1 s.d. increase in CT was associated with 0.39 s.d. and 0.26 s.d. increase in between-person threat appraisal and negative affect, respectively. Furthermore, greater daily threat appraisal predicted greater daily negative affect in the evening (estimate = 0.40, s.e. = 0.13, 95% CI 0.19–0.60, *p* = 0.002). Finally, both greater daily threat appraisals (estimate = 0.49, s.e. = 0.11, 95% CI 0.32–0.67, *p* < 0.001) and negative affect (estimate = 0.37, s.e. = 0.10, 95% CI 0.20–0.53, *p* < 0.001) predicted greater depressive symptoms. The direct path between reported CT and depressive symptoms was no longer significant (estimate = −0.09, s.e. = 0.08, 95% CI −0.22 to 0.04, *p* = 0.26), indicating mediation by daily stress processes. Mediation was supported by the total indirect effect from reported CT to depressive symptoms through daily stress processes (estimate = 0.34, s.e. = 0.08, 95% CI 0.22–0.46, *p* < 0.001). Daily threat appraisals accounted for more than 50% of the total indirect effect from reported CT to depressive symptoms (Fig. 1c, indirect path 1; estimate = 0.19, s.e. = 0.07, 95% CI 0.08–0.30, *p* = 0.004). Post hoc analyses showed that lower daily threat appraisals buffered depressive symptoms in those who reported at least moderate CT severity (Fig. 2, panel *a*). A similar pattern was present in those who reported no/low CT severity (Fig. 2, panel *b*).

### Sensitivity analyses

Given that day-level data were nested within the study waves (baseline, 9-month, and 18-month follow-up), we also considered a three-level MSEM (level-1: between-day, level-2: between-wave, and level-3: between-person). However, there was a lack of between-wave variances for threat appraisal (7%), negative affect (7%), and depressive symptoms (29%), so we only presented findings from the level-2 model (though between-person findings from the level-3 MSEM were consistent with findings from the level-2 MSEM). Furthermore, we also considered an alternative level-2 model with depressive symptoms as the mediator and daily stress processes as the outcome. The model fit indices for this alternative model are significantly worse than the hypothesized model (Table 2).

In addition, we tested whether significant MSEM results remained after controlling for current overall perceived stress in the past month (PSS) at the time of CT recall. Reported CT remained significantly associated with daily threat appraisals (estimate = 0.42, s.e. = 0.10, 95% CI 0.26–0.58, *p* < 0.001) and daily negative affect (estimate = 0.27, s.e. = 0.11, 95% CI 0.09–0.45, *p* = 0.016). Furthermore, daily threat appraisals remained significantly associated with daily negative affect (estimate = 0.39, s.e. = 0.13, 95% CI 0.18–0.60, *p* = 0.002), and both daily threat appraisals (estimate = 0.28, s.e. = 0.10, 95% CI 0.12–0.44, *p* = 0.005) and daily negative affect (estimate = 0.30, s.e. = 0.11, 95% CI 0.12–0.48, *p* = 0.007) remained significantly associated with depressive symptoms. Adding PSS into the model attenuated the indirect effect from reported CT to depressive symptoms through daily stress processes, though a significant indirect effect prevailed (estimate = 0.25, s.e. = 0.07, 95% CI 0.14–0.35, *p* < 0.001). Similarly, specific indirect pathways through daily threat appraisals (estimate = 0.12, s.e. = 0.05, 95% CI 0.03–0.20, *p* = 0.023), daily negative affect (estimate = 0.08, s.e. = 0.04, 95% CI 0.01–0.15, *p* = 0.042), and both daily threat appraisals and daily negative affect (estimate = 0.05, s.e. = 0.03, 95% CI 0.01–0.09, *p* = 0.044) were slightly attenuated but remained significant. The indirect effect through daily threat appraisals still accounted for around 50% of the indirect effect from reported CT to depressive symptoms even after controlling for current perceived stress in the past month.

Lastly, we conducted exploratory models within caregivers and controls separately. Overall, findings showed a pattern that was highly consistent with the main findings, though some associations became non-significant, as expected given the much smaller sample size in each group (for details, please see online Supplementary Analysis S1 and S2; Figures S4 and S5; Tables S2 and S3).

## Discussion

The study examined daily cognitive-affective stress processes as a risk pathway linking CT to depressive symptoms in midlife. Results showed that reported CT predicted elevated depressive symptoms. Daily threat appraisals and negative affect in the evening mediated the effect of reported CT on depressive symptoms. Particularly daily threat appraisals, which explained 50% of the total indirect effect from CT to depressive symptoms, may play a key role in molding depression risk.

Our data suggest that reported CT can leave ‘scars’ on adult mental health even in a healthy sub-clinical population. This replicates prior evidence of increased risk for depression (Danese & Baldwin, 2017; Green et al., 2010; Kendler et al., 2000; Kessler et al., 2010; Repetti, Taylor, & Seeman, 2002). It is notable that almost 80% of individuals with reported CT also reported at least mild depressive symptoms. The mental health risks may be conferred by the pervasive biopsychosocial effects (Danese & Lewis, 2017; Miller et al., 2011; Nelson, 2017; Shalev, 2012; Shonkoff & Garner, 2012) that trauma may have during periods in which the brain and physiological systems develop.

This study sheds light on the role of daily psychological stress processes as a persistent risk pathway. Reported CT predicted greater threat appraisals and negative affect in response to daily minor events, consistent with prior studies (Glaser et al., 2006; Infurna et al., 2015; Kong et al., 2019; Lardinois et al., 2011; Mallers et al., 2010; Weltz et al., 2016). This suggests that reported CT can have long-lasting effects on individuals’ mental filter – how they interpret and affectively respond to everyday hassles. In turn, maladaptive daily stress responses predicted elevated depressive symptoms, replicating prior studies on increased affective reactivity and mental health (Charles et al., 2013; Cohen et al., 2005) and demonstrating in an ecological context that daily threat appraisals are associated with elevated depressive symptoms.

That CT predicts poorer mental health in general is not new. But determining how, on a daily basis, this is working, is novel. We were able to directly test mediation in our study of daily stress processes. Results showed that greater reported CT predicted elevated depressive symptoms through greater daily threat appraisals and greater daily negative affect. Notably, more than half of this indirect effect on depressive symptoms was explained by how individuals appraised everyday stressors as having a greater negative impact on their lives. Post hoc analyses showed that greater threat appraisals were associated with elevated depressive symptoms in participants who reported at least moderate CT severity; conversely, lower threat appraisals were linked with lower depressive symptoms – to the extent that scores were asymptomatic and comparable to participants who reported no/low CT severity. A similar gradient relationship between threat appraisals and depressive symptoms was also found in participants with no/low reported CT severity. These data suggest that daily threat appraisals constitute a risk and resilience factor for those with and without a reported history of CT and may provide a promising target for depression interventions.

### Limitations

We did not recruit a sample with known depression, so conclusions are limited to a sub-clinical sample of participants at risk for depression. Furthermore, the study sample was highly selective with eligibility criteria and sociodemographic characteristics that are not representative of the general US population or the majority of women with a history of CT. Thus, results are likely only relevant for white women of higher SES (the majority of the sample has a bachelor's degree and an annual household income ⩾$ 100 000) with no major diseases or serious psychiatric disorders – factors that confer resilience (Almeida, 2005; Chui, Hay, & Diehl, 2012). For this reason, findings may only apply to women who tend to be white and of higher socioeconomic status with a reported history of CT. It will be important to test these relationships in samples that are lower income and more racially and ethnically diverse. Other stress-buffering and protective factors, such as current or past mental health treatment (e.g. CBT, supportive therapy or resources to cope with daily challenges) also warrant investigation in future studies. Conclusions are further limited to the specific characteristics of the sample that included mothers across the chronic stress spectrum (both mothers of children with an autism spectrum disorder as well as mothers of neurotypical children). The effects of chronic caregiver stress on daily stress perceptions and mood states are described elsewhere (Crosswell et al., 2020), and our results shed some light on the specific effects of reported CT. Caregivers and controls had different selection criteria (e.g. differences in PSS criteria; current major depression and antidepressant use permitted among caregivers) and also differed in two of the five types of CT (caregivers had higher scores on emotional abuse and physical neglect compared to controls), raising the possibility that findings are confounded by caregiver group status. However, all main models controlled for caregiver group status and exploratory models within caregivers and controls separately yielded an overall pattern of effects that was highly consistent with the main findings. Experiencing both childhood and adult (caregiver) stress might be associated with increased depression risk. However, interaction effects were not examined due to lack of power. Nevertheless, the effects of reported CT were present above and beyond chronic caregiver stress in adulthood, highlighting the role of the early environment in shaping daily stress responses and mental health in midlife.

Another limitation is that CT was assessed via retrospective self-report with the CTQ, which could be affected by reporting/recall biases linked, for example, to current perceived stress at the time of recall (Baldwin, Reuben, Newbury, & Danese, 2019; Danese, 2020; Dube, Williamson, Thompson, Felitti, & Anda, 2004; Maughan & Rutter, 1997). Thus, there is the possibility that the CTQ may measure negative biases in autobiographical memory or current affect and stress states rather than actual adversity exposure, which, in turn, may predict depressive symptoms via persistence of negative cognitive-affective biases in daily stress processes (Danese, 2020). Thus, it is conceivable that the findings, which were based on self-report data, do not reflect the ‘long shadow of CT’ but rather correlates of unhelpful cognition-affective states about the self and the environment that are related to the ‘subjective experience’ of CT (Danese & Widom, 2020). Whether the CTQ captures the long-term consequences or the subjective experience of CT, a greater understanding of the underlying mechanisms is important to explain its association with psychopathology and inform treatment development (Nanni, Uher, & Danese, 2012). To at least partly account for such potential cognitive-affective biases as in previous research (Danese & Widom, 2020), we conducted sensitivity analyses that adjusted for overall perceived stress at baseline – a proxy measure for capturing negative cognitive-affective states at the time of CT recall. Notably, findings were not altered adjusting for overall perceived stress, strengthening our interpretation of findings. Another limitation was that daily diaries only retrospectively assessed threat appraisals in the evening, not concurrently. Though shorter time windows minimize retrospective biases (Ebner-Priemer & Trull, 2009), future studies will benefit from real-time ecological assessments. Lastly, due to the lack of variability between study assessments, we could not capture how individual changes in daily stress processes related to temporal changes in depressive symptoms. Our results thus examine between-person associations, showing stable, trait-like relationships between CT, daily stress response habits, and depressive symptoms.

### Implications

How people respond each day matters. Developing more adaptive daily stress responses, particularly lowering threat appraisals in safe environmental contexts, may foster resilience. Since threat appraisals are often made quickly and on ‘autopilot’, awareness is a prerequisite. Appraisals that emphasize physical and emotional safety may be particularly salient for individuals with CT (Gilbert, McEwan, Matos, & Rivis, 2011). Mindfulness-based practices facilitate both present moment awareness and re-appraisals of stressors as being benign (Garland, Geschwind, Peeters, & Wichers, 2015; Garland, Kiken, Faurot, Palsson, & Gaylord, 2017), providing a promising intervention approach. Appraisals are also shaped by past experiences that are encoded into autobiographical memories and carried forward as narratives that shape reality (McCrory et al., 2017). Traumatic experiences fuel threat narratives. Processing traumatic memories and creating new narratives are key components of trauma-focused interventions (Cohen, Mannarino, Kliethermes, & Murray, 2012; Ehlers, 2013), which might have positive downstream effects for stress appraisals.

### Summary

A reported history of CT casts a long shadow into later adult life by shaping the lens through which everyday events are interpreted and experienced, creating an underlying stress vulnerability that has important mental health consequences even in sub-clinical populations. Daily psychological stress processes, especially threat appraisals, serve as promising, malleable intervention targets for individuals with reported CT.

## Supplementary material

The supplementary material for this article can be found at https://doi.org/10.1017/S0033291721000921
