# Supplementary material for: The long shadow of childhood trauma for depression in midlife: examining daily psychological stress processes as a persistent risk pathway
Source: Psychol Med. 2021 Mar 26;52(16):4029–38. Online ahead of print. doi: 10.1017/S0033291721000921 (PMC8647837; doi:10.1017/S0033291721000921)
Supplement: Supplementary file 1 [file S0033291721000921sup001.docx]

**Online Supplementary Materials**

**Table of Contents**

**Supplemental Figure 1.** **Changes of depressive symptoms across the 1.5-year period.**

**Supplemental Figure 2.** **Hypothesized model.**

**Supplemental Figure 3. Alternative model.**

**Supplemental Table 1. Unstandardized and standardized** **results from the multilevel structural equation modeling.**

**Supplemental Analysis 1. Among Caregivers (n = 92).**

**Supplemental Figure 4. Findings from the prediction of depressive symptoms by reported childhood trauma among caregivers.**

**Supplemental Table 2. Model fit indices for the analyses among caregivers.**

**Supplemental Analysis 2. Among Maternal Controls (n = 91).**

**Supplemental Figure 5. Findings from the prediction of depressive symptoms by reported childhood trauma among maternal controls.**

**Supplemental Table 3. Model fit indices for the analyses among maternal controls.**

**
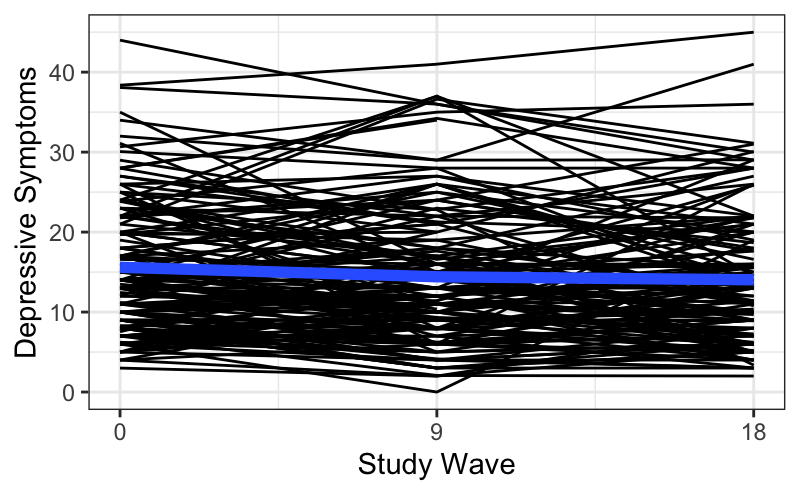
**

**Supplemental Figure 1.** **Changes of depressive symptoms across the 1.5-year period.** Depressive symptoms across study waves were relatively stable. Mean depressive symptoms at baseline were 15.61 (*SD* = 8.07). The means for 9-month and 18-month follow-up were 14.41 (*SD* = 8.73) and 14.04 (*SD* = 8.53), respectively.

**(A)**

Depressive Symptoms

**(B)**

Daily Threat Appraisal (WP)

Daily Negative Affect (WP)

Between-level

Within-level

Depressive Symptoms

**(C)**

Depressive Symptoms

Indirect path 1

Depressive Symptoms

Indirect path 2

Depressive Symptoms

Indirect path 3

**Supplemental Figure 2.** **Hypothesized model.**

(A) A structural equation model (SEM) in which reported childhood trauma predicts depressive symptoms.

(B) Multilevel structural equation model (MSEM) in which the association between reported childhood trauma and depressive symptoms is mediated by daily stress processes (BP = between-person; WP = within-person).

(C) Three possible indirect paths through which reported childhood trauma predicts depressive symptoms (all indirect paths were in the between-level). Note: squares = observed variables; circles = latent variables.


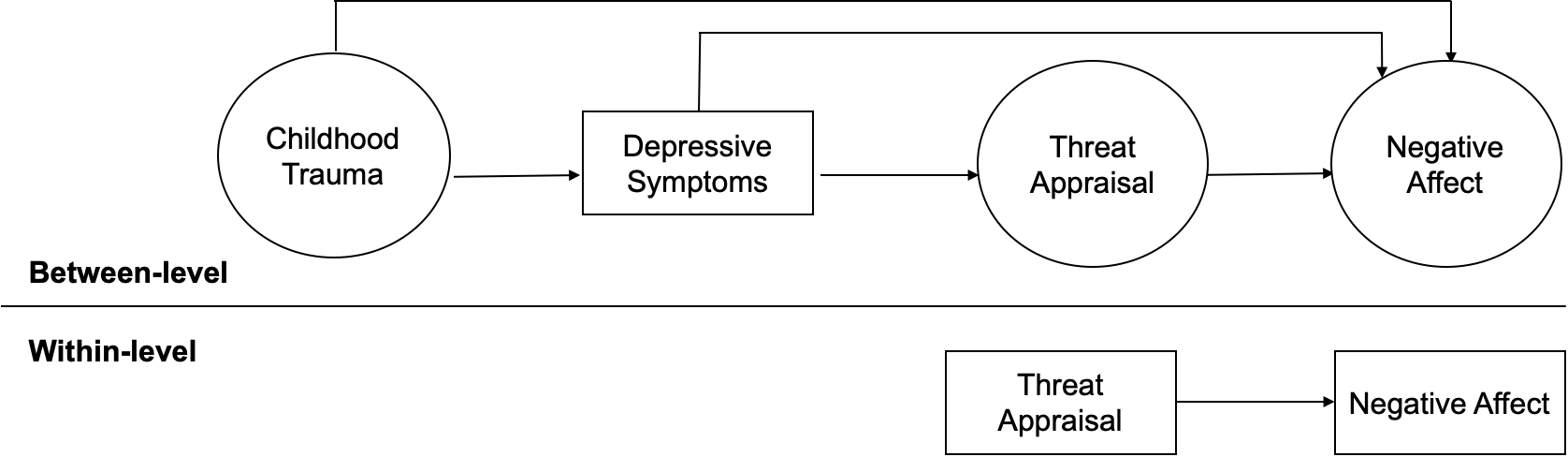


**Supplemental Figure 3. Alternative model.** In this alternative model depressive symptoms mediate the association between childhood trauma and daily stress processes.

**Supplemental Table 1. Unstandardized and standardized** **results from the multilevel structural equation modeling.**

| Predictor | Outcome | | | | | |
| --- | --- | --- | --- | --- | --- | --- |
|  | Threat Appraisal | | Negative Affect | | Depressive Symptoms | |
|  | Unstandardized Model | Standardized Model | Unstandardized Model | Standardized Model | Unstandardized Model | Standardized Model |
| **Within-level** |  |  |  |  |  |  |
| Threat Appraisal (TA) |  |  | 0.12 (0.01) ^***^ | 0.28 (0.02) ^***^ |  |  |
|  |  |  |  |  |  |  |
| **Between-level** |  |  |  |  |  |  |
| Direct |  |  |  |  |  |  |
| Childhood Trauma (CT) | 0.06 (0.02) ^***^ | 0.39 (0.09) ^***^ | 0.03 (0.01) ^*^ | 0.26 (0.11) ^*^ | -0.21 (0.18) | -0.09 (0.08) |
| Threat Appraisal (TA) |  |  | 0.25 (0.08) ^**^ | 0.40 (0.13) ^**^ | 7.04 (1.62) ^***^ | 0.49 (0.11) ^***^ |
| Negative Affect (NA) |  |  |  |  | 8.22 (2.23) ^***^ | 0.37 (0.10) ^***^ |
| Indirect |  |  |  |  |  |  |
| CT 🡪 TA 🡪 Depressive Symptoms |  |  |  |  | 0.45 (0.17) ^**^ | 0.19 (0.07) ^**^ |
| CT 🡪 NA 🡪 Depressive Symptoms |  |  |  |  | 0.22 (0.10) ^*^ | 0.09 (0.05) ^*^ |
| CT 🡪 TA 🡪 NA 🡪 Depressive Symptoms |  |  |  |  | 0.13 (0.06) ^*^ | 0.06 (0.03) ^*^ |
| Total indirect effect |  |  |  |  | 0.79 (0.19) ^***^ | 0.34 (0.08) ^***^ |

Note: Values in parentheses indicate standard errors; *, *p* < .05; **, *p* < .01, ***, *p* < .001.

**Supplemental Analysis 1. Among Caregivers (*n* = 92; Standardized Results Reported).**

The structural equation model among caregivers indicated that greater reported CT predicted higher depressive symptoms over the 1.5-year period (Supplemental Figure 4A; estimate = 0.30, *SE* = .11, 95% CI 0.12 to 0.47, *p* = .006), controlling for age, marital status, education, and annual household income. The MSEM findings indicated that the majority of the variances for threat appraisal (78%) and negative affect (61%) were within-day, supporting the need to consider the nesting data. Adding daily stress processes into the model improved the goodness of fit criteria (see Supplemental Table 2). Findings from Level 1 (Supplemental Figure 4B; within-level) indicated that on a day in which participants reported greater threat appraisals, they also reported greater negative affect in the evening (estimate = 0.27, *SE* = 0.02, 95% CI 0.23 to 0.30, *p* < .001), controlling for between-person differences in exposure to at least moderately severe stress days (objective severity codes from independent raters).

Results from Level 2 (Supplemental Figure 4B, between-level; controlling for age, marital status, education, income, and between-person differences in exposure to at least moderately severe stress days) indicated that greater reported CT predicted greater daily threat appraisals (estimate = 0.45, *SE* = 0.12, 95% CI 0.25 to 0.64, *p* < .001), but not negative affect (estimate = 0.17, *SE* = 0.14, 95% CI -0.06 to 0.40, *p* = .22). Furthermore, greater daily threat appraisal predicted greater daily negative affect in the evening (estimate = 0.54, *SE* = 0.13, 95% CI 0.33 to 0.75, *p* < .001). Finally, both greater daily threat appraisals (estimate = 0.43, *SE* = 0.14, 95% CI 0.20 to 0.66, *p* =.002) and negative affect (estimate = 0.36, *SE* = 0.14, 95% CI 0.12 to 0.59, *p* = .013) predicted greater depressive symptoms. The direct path between reported CT and depressive symptoms was no longer significant (estimate = -0.06, *SE* = 0.11, 95% CI -0.25 to 0.12, *p* = .58), indicating mediation by daily stress processes. Mediation was supported by the total indirect effect from reported CT to depressive symptoms through daily stress processes (estimate = 0.34, *SE* = 0.09, 95% CI 0.19 to 0.49, *p* < .001). Daily threat appraisals accounted for more than 50% of the total indirect effect from reported CT to depressive symptoms (Supplemental Figure 4C, Indirect path 1; estimate = 0.19, *SE* = 0.09, 95% CI 0.04 to 0.34, *p* = .04).

**Sensitivity analysis.** We considered an alternative model with depressive symptoms as the mediator and daily stress processes as the outcome. The model fit indices for this alternative model are significantly worse than the hypothesized model (Supplemental Table 2). We also tested whether significant MSEM results remained after controlling for current overall perceived stress in the past month (PSS) at the time of CT recall. Reported CT remained significantly associated with daily threat appraisals (estimate = 0.46, *SE* = 0.13, 95% CI 0.26 to 0.67, *p* < .001). The association between CT report and daily negative affect remained non-significant (estimate = 0.17, *SE* = 0.14, 95% CI -0.06 to 0.40, *p* = .21). Furthermore, daily threat appraisals remained significantly associated with daily negative affect (estimate = 0.54, *SE* = 0.13, 95% CI 0.33 to 0.75, *p* < .001). Daily threat appraisals was marginally associated with depressive symptoms (estimate = 0.23, *SE* = 0.13, 95% CI 0.02 to 0.44, *p* = .079), while daily negative affect (estimate = 0.34, *SE* = 0.15, 95% CI 0.09 to 0.60, *p* = .025) remained significantly associated with depressive symptoms. Adding PSS into the model attenuated the indirect effect from reported CT to depressive symptoms through daily stress processes, though a significant indirect effect prevailed (estimate = 0.25, *SE* = 0.08, 95% CI 0.12 to 0.38, *p* = .002). Specific indirect pathways through daily threat appraisals (estimate = 0.11, *SE* = 0.07, 95% CI -0.01 to 0.22, *p* = .13), daily negative affect (estimate = 0.06, *SE* = 0.05, 95% CI -0.03 to 0.15, *p* = .25), and both daily threat appraisals and daily negative affect (estimate = 0.09, *SE* = 0.05, 95% CI 0.01 to 0.16, *p* = .063) became non-significant although the directions of the mediations remained consistent.

**(A)**

Depressive Symptoms

0.30 (0.11)

Daily Threat Appraisal

Daily Negative Affect

Between-level

Within-level

Depressive Symptoms

0.45 (0.12)

0.54 (0.13)

0.36 (0.14)

0.17 (0.14)

0.43 (0.14)

0.27 (0.02)

-0.06 (0.11)

**(B)**

Depressive Symptoms

**(C)**

Indirect path 1 was significant (estimate = 0.19, *SE* = 0.09, 95% CI 0.04 to 0.34, *p* = .04)

Depressive Symptoms

Indirect path 2 was not significant (estimate = 0.06, *SE* = 0.06, 95% CI -0.03 to 0.15, *p* = .27)

Depressive Symptoms

Indirect path 3 was significant (estimate = 0.09, *SE* = 0.04, 95% CI 0.02 to 0.15, *p* = .039)

**Supplemental Figure 4. Findings from the prediction of depressive symptoms by reported childhood trauma among caregivers.**

**Supplemental Table 2. Model fit indices for the analyses among caregivers.**

| Model | χ^2^ | df | χ^2^/df | RMSEA | CFI | TLI | SRMR-W | SRMR-B | AIC | BIC | a-BIC |
| --- | --- | --- | --- | --- | --- | --- | --- | --- | --- | --- | --- |
| *Childhood Trauma, Daily Stress Processes, and Depressive Symptoms* | | | | | | | | | | | |
| SEM | 67.31 | 36 |  | .098 | .79 | .71 | .10^a^ | | 4652.5 | 4750.0 | 4626.9 |
| Hypothesized MSEM | 90.98 | 52 |  | .02 | .93 | .90 | .00 | .09 | 12561.5 | 12868.8 | 12684.6 |
| Alternative MSEM^b^ | 96.9 | 52 |  | .02 | .92 | .88 | .00 | .10 | 12567.6 | 12869.7 | 12688.6 |

Note: SEM = structural equation model; MSEM = multilevel structural equation model; χ^2^ = chi-square test of model fit; df = degrees of freedom for the chi-square test of model fit; RMSEA = root mean square error of approximation; CFI = comparative fit index; TLI = Tucker–Lewis index; SRMR-B = standardized root mean square residual for between level; SRMR-W = standardized root mean square residual for within level; AIC = Akaike’s information criterion; BIC = Bayesian information criterion; a-BIC = sample-size adjusted Bayesian information criterion. ^a^The structural equation model only has one value of standardized root mean square residual.

^b^ Alternative MSEM with depressive symptoms as mediator.

**Supplemental Analysis 2. Among Maternal Controls (*n* = 91; Standardized Results Reported).**

The structural equation model among maternal controls indicated that greater reported CT predicted higher depressive symptoms over the 1.5-year period (Supplemental Figure 5A; estimate = 0.23, *SE* = .11, 95% CI 0.05 to 0.41, *p* = .035), controlling for age, marital status, education, and annual household income. The MSEM findings indicated that the majority of the variances for threat appraisal (81%) and negative affect (69%) were within-day, supporting the need to consider the nesting data. Adding daily stress processes into the model improved the goodness of fit criteria (see Supplemental Table 3). Findings from Level 1 (Supplemental Figure 5B; within-level) indicated that on a day in which participants reported greater threat appraisals, they also reported greater negative affect in the evening (estimate = 0.30, *SE* = 0.03, 95% CI 0.26 to 0.35, *p* < .001), controlling for between-person differences in exposure to at least moderately severe stress days (objective severity codes from independent raters).

Results from Level 2 (Supplemental Figure 5B, between-level; controlling for age, marital status, education, income, and between-person differences in exposure to at least moderately severe stress days) indicated that greater reported CT was not significantly associated with daily threat appraisals (estimate = 0.20, *SE* = 0.12, 95% CI -0.01 to 0.40, *p* = .103) or negative affect (estimate = 0.26, *SE* = 0.14, 95% CI 0.03 to 0.50, *p* = .065), though effects were trending in the same direction as findings in the combined sample. Greater daily threat appraisal significantly predicted greater daily negative affect in the evening (estimate = 0.39, *SE* = 0.10, 95% CI 0.23 to 0.55, *p* < .001). Finally, both greater daily threat appraisals (estimate = 0.73, *SE* = 0.32, 95% CI 0.20 to 1.26, *p* =.025) and negative affect (estimate = 0.39, *SE* = 0.13, 95% CI 0.17 to 0.60, *p* = .003) predicted greater depressive symptoms. The direct path between reported CT and depressive symptoms was no longer significant (estimate = -0.17, *SE* = 0.12, 95% CI -0.36 to 0.03, *p* = .164), indicating mediation by daily stress processes. Mediation was supported by the total indirect effect from reported CT to depressive symptoms through daily stress processes (estimate = 0.27, *SE* = 0.09, 95% CI 0.12 to 0.43, *p* = .003).

**Sensitivity analysis.** We considered an alternative model with depressive symptoms as the mediator and daily stress processes as the outcome. The model fit indices for this alternative model are significantly worse than the hypothesized model (Supplemental Table 3). We also tested whether significant MSEM results remained after controlling for current overall perceived stress in the past month (PSS) at the time of CT recall. The non-significant marginal associations between reported CT and daily threat appraisals (estimate = 0.23, *SE* = 0.13, 95% CI 0.02 to 0.44, *p* = .078) or negative affect (estimate = 0.26, *SE* = 0.14, 95% CI 0.03 to 0.50, *p* = .061) remained. Daily threat appraisals remained significantly associated with daily negative affect (estimate = 0.39, *SE* = 0.10, 95% CI 0.23 to 0.54, *p* < .001). Daily threat appraisals was not associated with depressive symptoms (estimate = 0.30, *SE* = 0.19, 95% CI -0.01 to 0.61, *p* = .114), while daily negative affect (estimate = 0.27, *SE* = 0.14, 95% CI 0.03 to 0.50, *p* = .062) was marginally associated with depressive symptoms. Adding PSS into the model attenuated the indirect effect from reported CT to depressive symptoms through daily stress processes, though a significant indirect effect prevailed (estimate = 0.16, *SE* = 0.08, 95% CI 0.03 to 0.29, *p* = .047). Specific indirect pathways through daily threat appraisals (estimate = 0.07, *SE* = 0.06, 95% CI -0.03 to 0.17, *p* = .26), daily negative affect (estimate = 0.07, *SE* = 0.06, 95% CI -0.02 to 0.16, *p* = .21), and both daily threat appraisals and daily negative affect (estimate = 0.02, *SE* = 0.02, 95% CI -0.01 to 0.05, *p* = .21) remained non-significant although the directions of the mediations remained consistent.

Daily Threat Appraisal

Daily Negative Affect

Between-level

Within-level

Depressive Symptoms

0.20 (0.12)

0.39 (0.10)

0.39 (0.13)

0.26 (0.14)

0.73 (0.32)

0.30 (0.03)

-0.17 (0.12)

**(B)**

Depressive Symptoms

0.23 (0.11)

**(A)**

**(C)**

Depressive Symptoms

Indirect path 1 was not significant (estimate = 0.14, *SE* = 0.10, 95% CI 0.04 to 0.34, *p* = .141)

Depressive Symptoms

Indirect path 2 was not significant (estimate = 0.10, *SE* = 0.07, 95% CI -0.03 to 0.15, *p* = .131)

Depressive Symptoms

Indirect path 3 was not significant (estimate = 0.03, *SE* = 0.02, 95% CI 0.02 to 0.15, *p* = .182)

**Supplemental Figure 5. Findings from the prediction of depressive symptoms by reported childhood trauma among maternal controls.**

**Supplemental Table 3. Model fit indices for the analyses among maternal controls.**

| Model | χ^2^ | df | χ^2^/df | RMSEA | CFI | TLI | SRMR-W | SRMR-B | AIC | BIC | a-BIC |
| --- | --- | --- | --- | --- | --- | --- | --- | --- | --- | --- | --- |
| *Childhood Trauma, Daily Stress Processes, and Depressive Symptoms* | | | | | | | | | | | |
| SEM | 111.33 | 36 |  | .15 | .62 | .49 | .10^a^ | | 4454.8 | 4552.7 | 4429.6 |
| Hypothesized MSEM | 112.1 | 52 |  | .03 | .90 | .85 | .00 | .09 | 11826.9 | 12133.2 | 11952.1 |
| Alternative MSEM^b^ | 114.7 | 52 |  | .03 | .89 | .84 | .00 | .10 | 11830.9 | 12137.2 | 11956.1 |

Note: SEM = structural equation model; MSEM = multilevel structural equation model; χ^2^ = chi-square test of model fit; df = degrees of freedom for the chi-square test of model fit; RMSEA = root mean square error of approximation; CFI = comparative fit index; TLI = Tucker–Lewis index; SRMR-B = standardized root mean square residual for between level; SRMR-W = standardized root mean square residual for within level; AIC = Akaike’s information criterion; BIC = Bayesian information criterion; a-BIC = sample-size adjusted Bayesian information criterion. ^a^The structural equation model only has one value of standardized root mean square residual.

^b^ Alternative MSEM with depressive symptoms as mediator.
